# Exposing the obscured influence of state-controlled media via causal inference of quotation propagation

**DOI:** 10.1038/s41598-024-78586-x

**Published:** 2025-01-07

**Authors:** Joseph Schlessinger, Richard Bennet, Jacob Coakwell, Steven Smith, Edward Kao

**Affiliations:** 1 MIT Lincoln Laboratory, 244 Wood Street, Lexington, MA 02421 US; 2 Accenture Federal Services, 800 North Glebe Road, Arlington, VA 22203 US

**Keywords:** Causal inference on networks, Network science, Intermedia-agenda setting, State-owned media influence, Narrative propagation, Influence operations, Complex networks, Scientific data, Computer science

## Abstract

Reporting by major media outlets influences news coverage by other outlets, resulting in an outsized impact on public opinion. Understanding this inter-outlet influence, known as intermedia agenda setting, is important for assessing the impact of state media and strategic communications. We demonstrate a novel method to quantify inter-outlet influence using causal inference on “quote following,” where one outlet uses the same quote as another outlet at a later date. By applying our methodology to a dataset of quotes from over 100,000 articles published in European media between May 2018 and October 2019, we reveal obscured influence by Russian state-affiliated media over other outlets and the general dynamics of European media. We find that Russian state-controlled media have a strong influence on the coverage of other Russian outlets, including independent outlets. Moreover, this influence extends to media ecosystems of other countries. Finally, we demonstrate quantifying inter-outlet influence on the specific topic of nuclear forces treaty, as an example of precision study of intermedia agenda setting on specific issues. Overall, our methodology quantifies sources and channels of influence between news outlets with important implications for strategic communication, influence operations, and media independence.

## Introduction

News coverage helps shape public opinion by “setting the agenda,” or affecting what issues the public considers^1^. The characteristics and effects of news propagation across social networks has important implications and has thus been the subject of considerable research^2,3^. As state actors fund and control international media outlets and attempt to influence topics to further their political agenda, the ability to detect and analyze such effects becomes increasingly important^4,5^. Though much attention has been given to government actors’ propaganda efforts, particularly in the case of Russia, the often-subtle ways in which state-affiliated outlets set the agenda for *other*mainstream news coverage remains obscured^6–8^.

Media outlets—including print, television, radio, and internet—continually influence each other. An influential outlet can affect how other outlets and the broader information environment treat a particular topic, determining the amount of coverage a topic receives and the sentiment in subsequent coverage. This inter-outlet influence is commonly referred to as *intermedia agenda setting*. An approach that measures the comparative influence of specific outlets over others can quantify how agendas are pushed across this information ecosystem and ultimately to the general public. Previous studies have taken a variety of approaches to measuring intermedia agenda setting. Some rely on instances where an article directly references a different outlet’s coverage^9^. Most studies rely on common article topics as the basic unit of analysis^10–14^. Nearly all of these studies use time series analysis of article topic volume to measure influence^15^. We improve upon previous work by using common quotes as our instrument for influence, which we argue provide more granularity and less noise compared to article topics. Moreover, we use a network causal inference framework to determine inter-outlet causal effects^16^.

Quotations play an important role in news articles. When an outlet features a quotation within an article, a decision is made by the authors to feature this particular voice. For instance, if a media outlet decided to predominately quote U.S. officials regarding the war in Ukraine, rather than Russian officials, that outlet would present a very different account to its audience than an outlet that relied solely on Russian official sourcing. The decisions made of whom to quote and how to frame those quotations form the core components of an outlet’s coverage of a news item or topic.

Outlets generally rely on other outlets’ coverage, to include their use of quotes, in line with the cascading network activation model^17,18^. The cascading network activation model is described as one where “media outlets ... create cascades of information among themselves, from, for example, more prestigious to less prestigious outlets”^19^. For example, news wires such as *Reuters*, *Agence France Presse*, and *TASS* have greater resources and access to sources than many other outlets, who often cite such wire services in their reporting. A common quote may indicate a cascade of information. Though it is possible that two outlets will share a quotation if both had journalists present at the same press conference, considering temporal differences in a quote’s appearance in coverage provides a good proxy for establishing the relationship between outlets. Furthermore, we can control for general newsworthiness by discounting quotes that are used by multiple outlets. Instances where only two outlets use a particular quote represent the greatest “signal” of potential influence. Though such instances may occasionally be coincidental, repeated instances of shared quotes over time are unlikely to be explained by coincidence alone.

We propose a novel quantitative approach to measure the effects in intermedia agenda setting and framing based on shared quotations within articles by different outlets. Using instances of *quote following*, where one outlet uses a quote in an article *after *another outlet, we can construct an influence network that reflects the baseline relationships between various outlets. Then, we can apply a network causal inference framework that measures outlets’ impacts on specific topics and sentiments^16,20^. For a more detailed explanation of the methodology, please refer to the Supplemental Materials. For this analysis, we examine influence patterns across a wide variety of geopolitical topics in European media with a particular focus on quotes that take a pro-Russia or pro-U.S. stance. We also demonstrate a case study on one particular topic: the Intermediate-Range Nuclear Forces (INF) Treaty. Applied to European and Russian media, our approach reveals the implicit ways that Russian state-controlled outlets influence other outlets. Though the state-controlled media exercise no direct authority over other outlets, they exert influence over which topics to report on and whom to quote on those topics^5,6^. The following results demonstrate our approach on a large, annotated dataset comprised of labeled quotes from a wide selection European and Russian public news media, containing 618,328 quotations from 123,396 articles published between May 2018 and October 2019. More information on the dataset can be found in the Supplemental Materials. Further, a partially-sanitized version of the dataset that is sufficient for replication can be found at the Mendeley Repository.

**Fig. 1:**
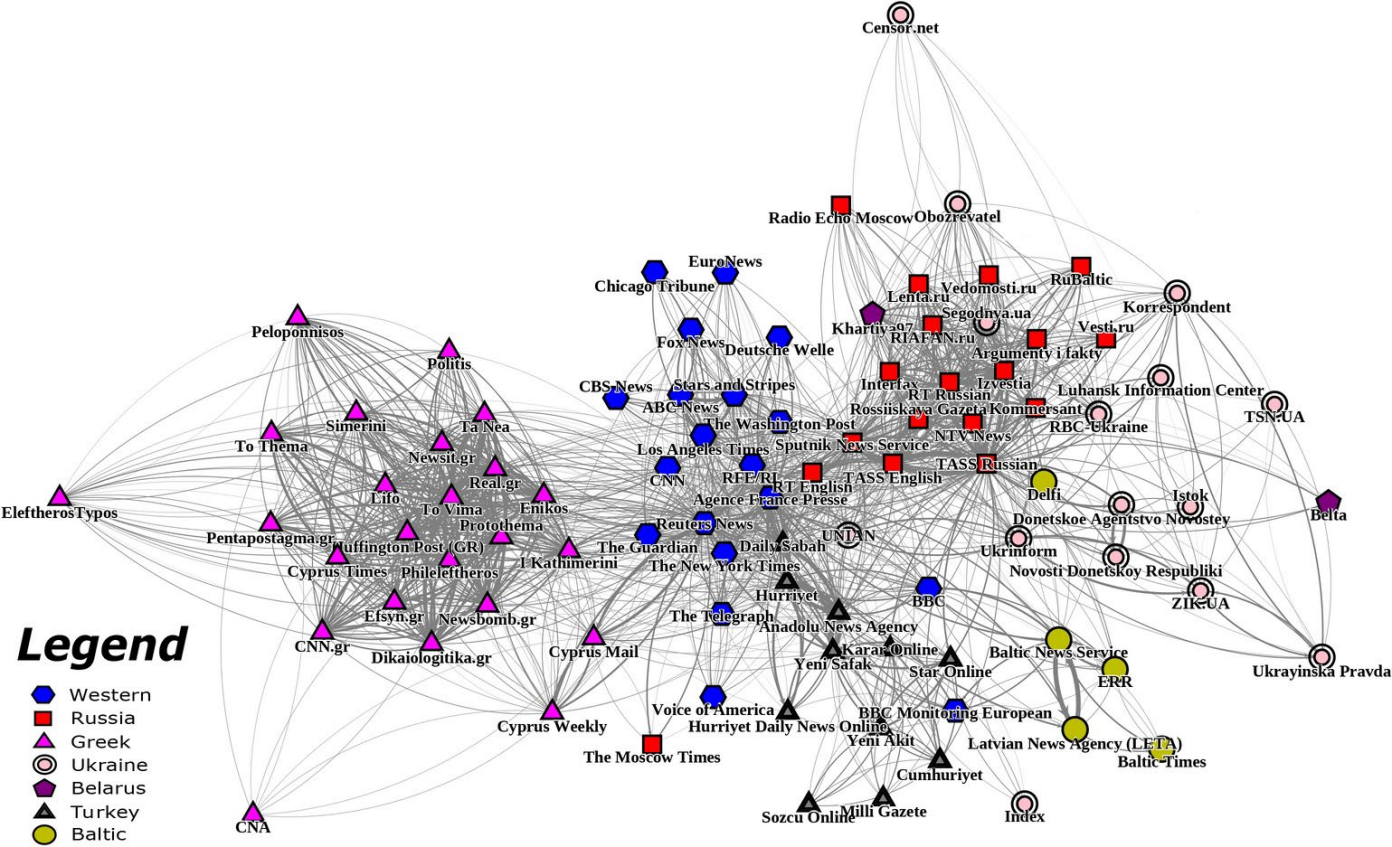
Constructed influence network of European media outlets. Edges represent past patterns of quote following with saliency weighting. Node locations were determined by a force-directed graph drawing algorithm^21^. A node’s shape and color each correspond to that news outlet’s country.

## Results

Focusing our analysis on the impact of state-controlled outlets on the overall media landscape, we analyze Russian state-controlled media and how it shapes media sentiment towards its competition with the U.S. Specifically, we process the quote data with either a pro-Russia (i.e. positive to Russia or negative to the U.S.) or a pro-U.S. (i.e. positive to the U.S. or negative to Russia) sentiment. For each media outlet, we estimate its causal impact with both pro-Russia and pro-U.S. quotes. Total impact represents the sum of pro-Russia and pro-U.S. impacts. In this paper, we define impact slant as the difference between an outlet’s pro-U.S. and pro-Russia impact scores, so a pro-Russia slant is negative and a pro-U.S. slant is positive.

Figure 1 depicts the baseline influence network constructed using quotes from all topics and sentiments. The influence network reveals distinctive geopolitical communities that emerge based on each outlets’ weighted edges. We have labeled the outlets based on their respective origin to emphasize this geopolitical structure within the influence network. These communities are primarily connected to each other by wire services like *Agence France Presse*, *UNIAN*, *Daily Sabah*, and *Sputnik News Service*, which straddle geographic boundaries. Although not a wire service, the English-language version of the Russian outlet *RT* (referred to as *RT English* in the network) is also located along the spine connecting the eastern Europe media environment to the Western media environment. There are some exceptions to strict geographic clustering, which can be explained by language and outlet orientation. For example, *The Moscow Times*, an English-language, independent media outlet, is separate from the main Russian community. The Russian outlet, *Radio Echo Moscow*, is removed from the main cluster of Russian media outlets due to its liberal orientation but nevertheless is closer to the cluster than *The Moscow Times* given its Russian-language reporting.

### Average outlet impact and slant

**Fig. 2:**
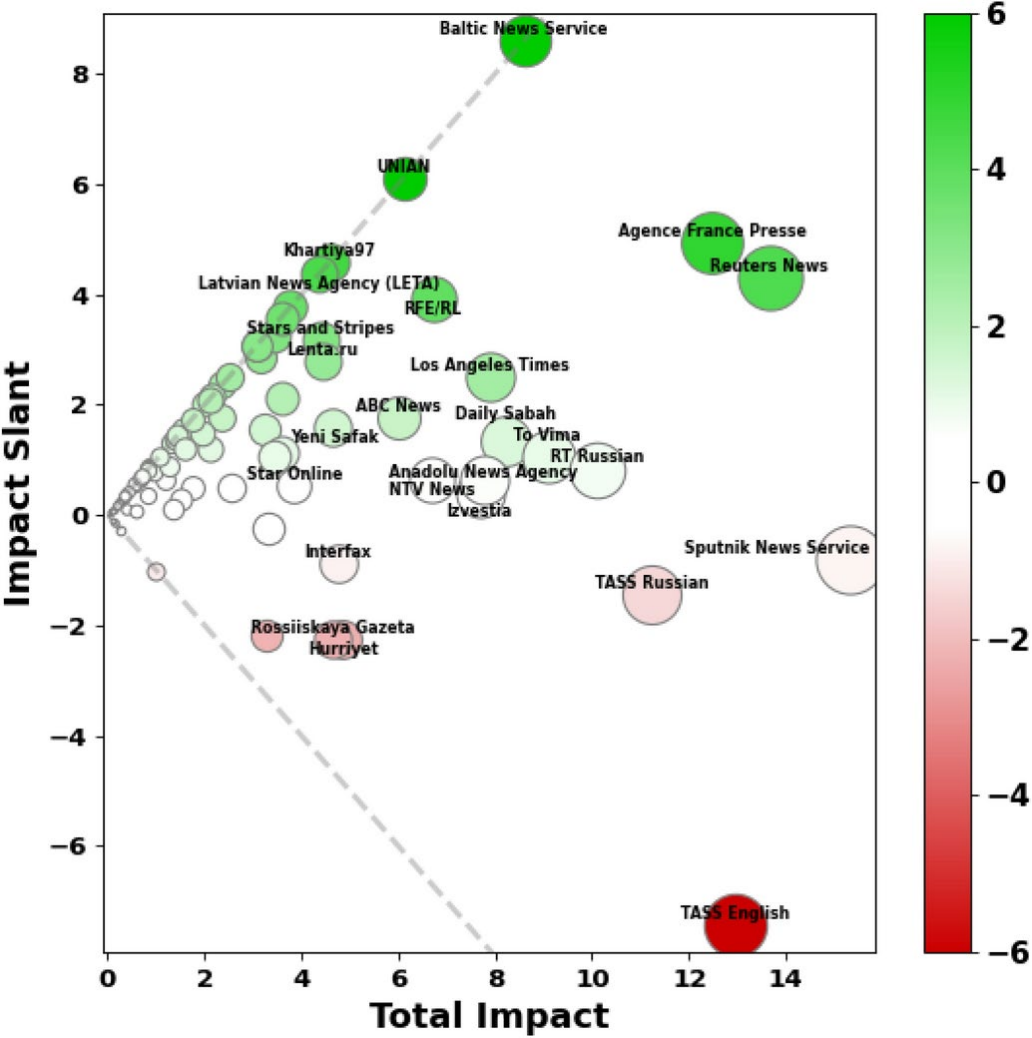
Media outlet impact slant versus total impact across all topics. For visual emphasis, outlets are also sized by total impact and colored by impact slant. Total impact represents the sum of pro-Russia and pro-U.S. impacts. Impact slant is the difference between an outlet’s pro-U.S. and pro-Russia impact scores, so a pro-Russia slant is negative, or red, and a pro-U.S. slant is positive, or green. Neutral outlets are white. The grey dotted lines indicate values where all impact came in either the pro-U.S. or pro-Russia slant.

In an analysis of the impact slant of outlets, we find that Western sources skew pro-U.S. while Russian sources skew pro-Russia. Figure 2 summarizes each outlet’s average total impact and impact slant across all topics. The majority of outlets are neutral overall. A few outlets, like *Baltic News Service*, *UNIAN*, and *Khartiya97*, exhibited only pro-U.S. impact, which suggests they almost exclusively publish quotes favorable to the U.S. and any outlets quoting from their articles are sharing pro-U.S. quotes. Most of the high impact outlets have a mixture of pro-Russia and pro-U.S. impact reflecting more balanced coverage.

Wire outlets like *Sputnik News Service*, *TASS*, *Reuters News*, and *Agence France Presse* have the highest impact level, which is consistent with their network centrality. It is also consistent with their increased article volume and role in “breaking” news stories. While impact slants largely match intuition, there are some surprises in the neutrality, and even slightly pro-U.S. slant, of certain Russian outlets. *RT Russian*, for example, skews slightly pro-U.S. We investigate some of these specific outlets in the following section.

### Examining individual outlet impacts

**Fig. 3:**
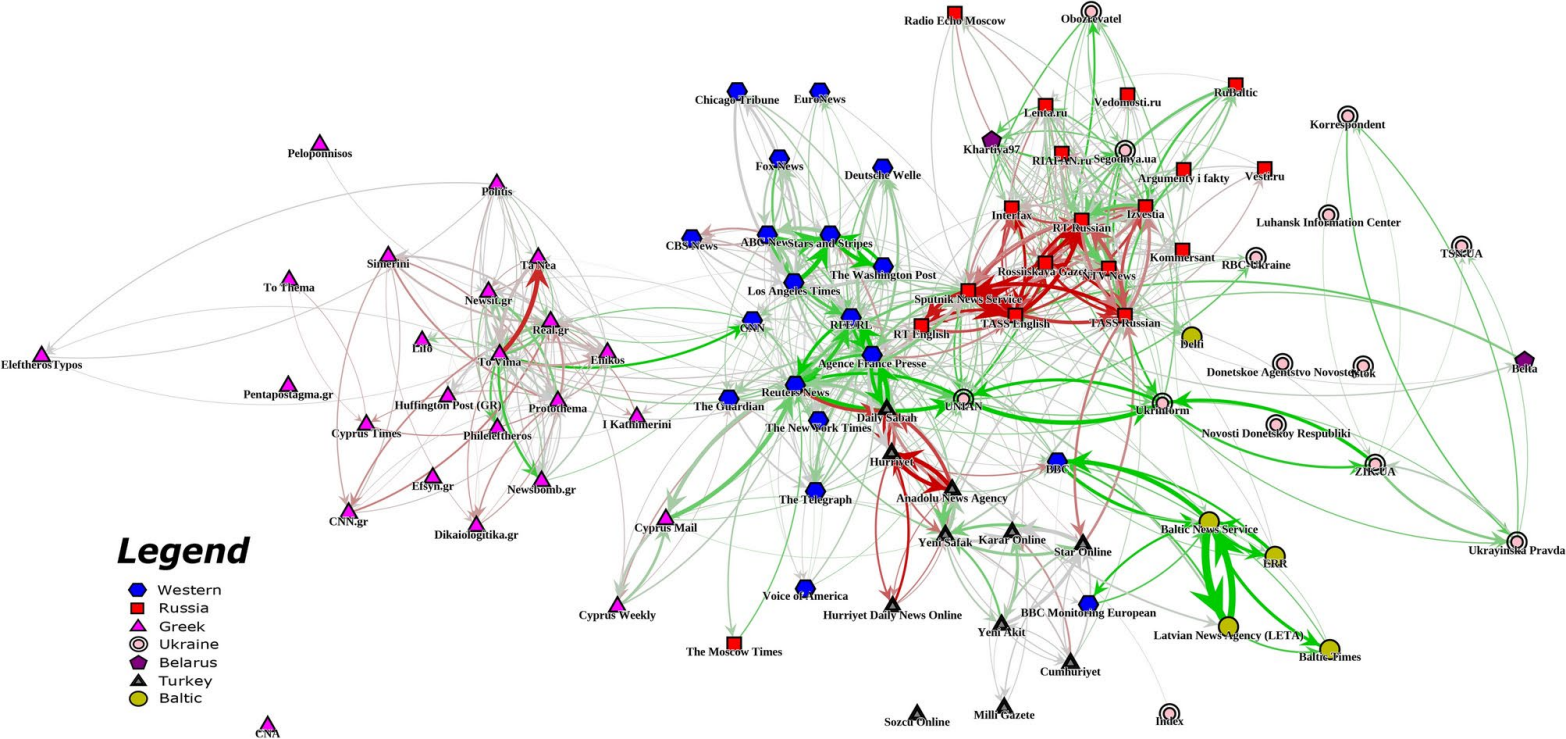
Impact network across all topics. Edges are sized according to total impact and colored according to impact slant. Red indicates pro-Russia impact slant, green indicates pro-U.S. impact slant, and grey indicates neutral impact. Node locations were determined by a force-directed graph drawing algorithm^21^. A node’s shape corresponds to that news outlet’s country.

**Fig. 4:**
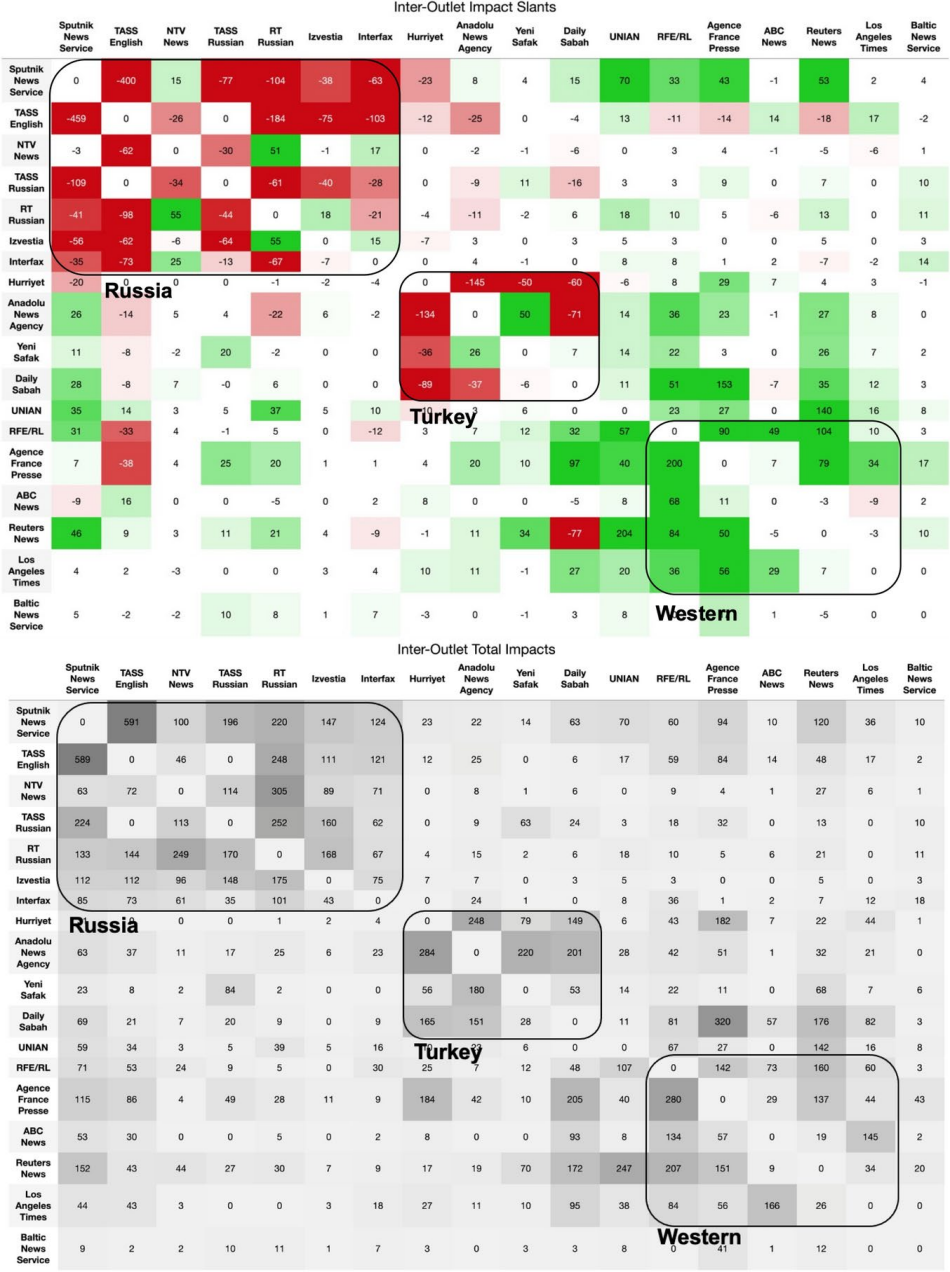
Impact slant (top) and total impact (bottom) of the 18 highest impact outlets. Refer to Figure 2 for more detailed definitions. The row represents the source outlet and column represents the impacted outlet. In the top table, cells are colored according to their slant, with pro-Russia being red and negative, neutral white and around zero, and pro-U.S. green and positive. In the bottom table, cells are shaded according to the magnitude of total impact.

Our methodology enables us to examine the impact of each outlet on other specific outlets. Figure 3 displays impacts as edges on the impact network, while Figure 4 summarizes the pairwise impacts of the most impactful outlets. Evident in the network community structure of the estimated causal impact between outlets, outlets typically influence others within their own geopolitical media ecosystem. This makes sense given language barriers between countries and the saliency of topics within a given country. Wire outlets, like *Reuters*, *AFP*, *Sputnik* and *TASS*, break this trend with significant impact reaching outside of their respective countries’ media environments. These news wires also have the greatest total impact across U.S. and European media environments. As the bridges of the estimated causal impact network, these wire outlets play the role of connecting the media ecosystems of different countries. Nearly all the pro-Russia impact is between Russian outlets and Turkish outlets; outside of Russia and Turkey, net impact is largely pro-U.S. Moreover, the imbalance in the directionality of the impact slant shows that outlets selectively follow quotes. For example, Western outlets follow the pro-U.S. quotes from Russian outlets, while Russian outlets follow the pro-Russia quotes from Western outlets.

Figure 3 and Figure 4 reveal a Russian echo chamber in which Russian state media outlets extensively borrow quotes from each other. Much of the net pro-Russia sentiment across European outlets comes from a few Russian outlets (*TASS*, *Sputnik*, *RT*). The volume of quote following among these outlets is much higher than any other outlet pairs. This may signify attempts by Russian state media to promote their narratives to the Russian audiences by boosting the messages of similarly state-controlled outlets.

Figure 4 also sheds light on *RT Russian* and *Izvestia*’s neutrality in impact slant. Compared to the other large Russian sources like *Sputnik* and *TASS*, *RT Russian* and *Izvestia* have much smaller impact scores. They do not seem to be as engaged in the Russian state media echo chamber. *Izvestia* and *RT Russian* even exert pro-U.S. net influences on each other, which can be attributed to the quotation of U.S. officials. Many of the most impactful quotes published by *RT Russian* are from U.S. officials criticizing Russia. Due to the editorial context surrounding the extracted quotes, these U.S. statements that are classified as “negative to Russia” often reflect negatively on the U.S. to the audiences of Russian media within the context of the whole article. For instance, when *RT Russian* quotes former U.S. Secretary of State Mike Pompeo saying that Russian violated the INF Treaty, commentary or a quote from the Kremlin follows, claiming the U.S. in fact violated the INF Treaty. Thus, as Russian media introduces some amount of U.S. statements for context setting purposes, audiences of Russian media may be conditioned to perceive U.S. statements as duplicitous or hypocritical. *RT Russian*’s overall pro-U.S. impact in fact indicates that the outlet is a common source of U.S. official statements for other Russian outlets. This finding is supported by the fact that *RT* has a well-established English-language service with access to press conferences or other English-language sources of U.S. official statements.

### State-affiliated media impacts

In the following section, we introduce two new concepts to our analysis. First, we work with the followings classifications of a media outlet: state-controlled, state-agenda, and independent. These classifications reflect to what extent the outlet’s state of origin controls the content being published in an outlet. State-agenda outlets, for instance, are categorized as not being directly controlled by the state, but as advancing the state agenda due to ownership or political ties to the state. Second, we present causal impact scores normalized by their potential outcomes, which allow us to compare impacts between outlets with different article outputs. For example, a normalized causal impact of 50% from *Sputnik* to *Izvestia* states we estimate that 50% of the instances *Izvestia* “follows” a quote from *Sputnik* are caused by *Sputnik* using the quote.

#### Obscured Russian state-affiliated media influence

**Fig. 5:**
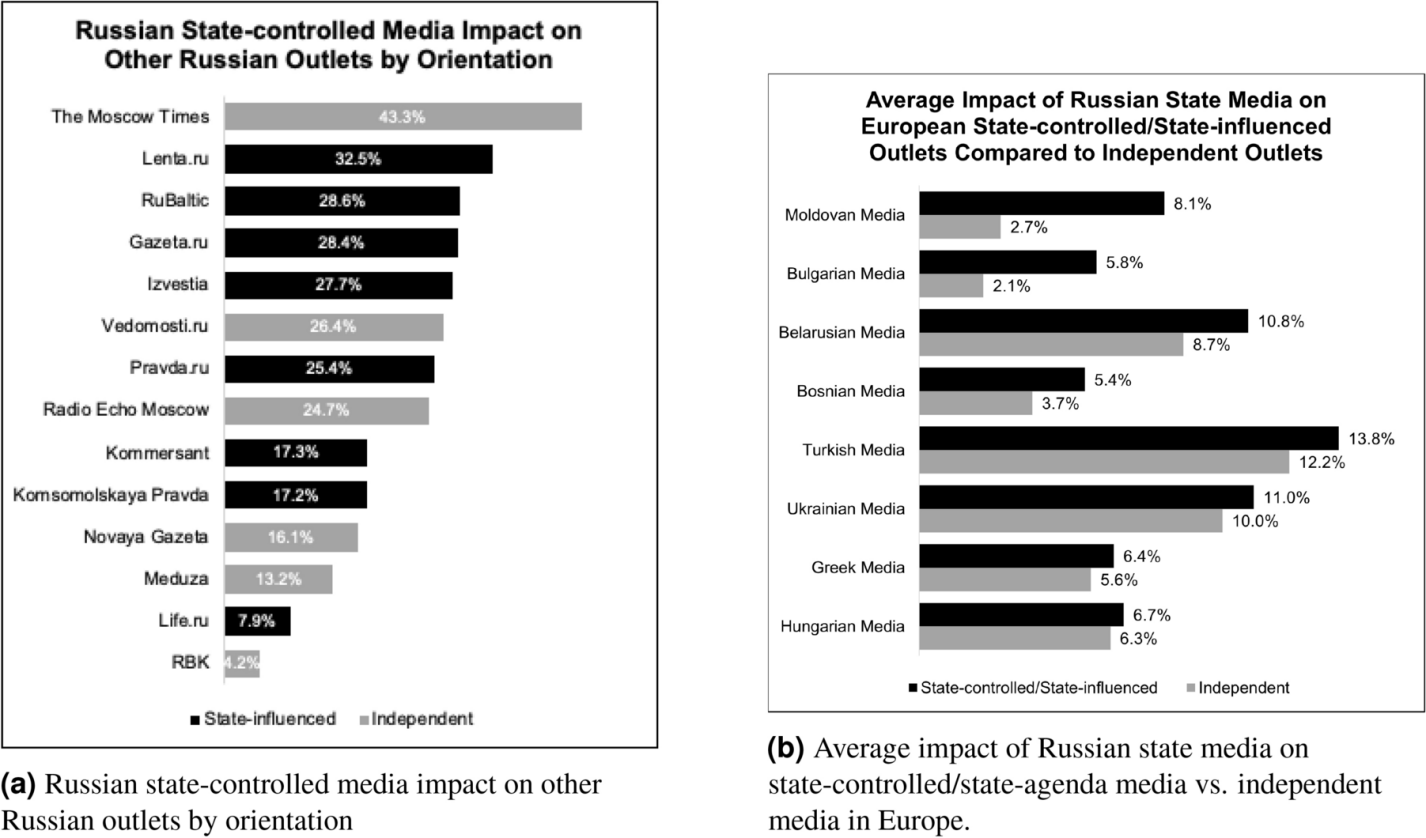
Impacts of Russian state media.

Our analysis reveals an obscured level of Russian influence, which would be of interest to any researchers of influence operations. As shown in Figure 5a, Russian state-controlled media have an average causal impact on 24.2% on all other Russian outlets. This is important to know because while an outlet may be independent, they nevertheless report on the same topics and use the same sources as Russian state media. *Radio Echo Moscow *exemplifies this condition with 24.7% of its quote following impacted by Russian state media. This outlet has previously been noted for its independence from the Russian state and often reports in liberal and pro-Western terms^22^. However, as shown in Figure 5a, state-controlled media frequently cause *Radio Echo Moscow* to use its quotes in its reporting. In other words, while *Radio Echo Moscow* is free from reporting in pro-Russian terms, the outlet is not free from reporting the agenda Russian state-controlled media has set.

#### Media independence as a limit on Russian state media influence

By dividing media outlets in each European country into state-affiliated (e.g. state-controlled and state-agenda) and independent, one can see in Figure 5b that on average Russian state media has greater influence on state-affiliated media than on independent media in all eight European countries in our dataset for which there were both state and independent outlets. This is possibly a result of countries without well-funded wire news services relying on the packaged reporting from news wires like Russian state-controlled *TASS*. These results may also indicate Russian political dominance, where state-controlled media of various countries are following Russian topics of interest with particular attention to official Russian sources. Regardless of the possible causes, the results of our analysis suggest media independence may decrease the impact that Russian state media has on the European media environment.

#### The impact of U.S. and Russian government-sponsored media in Europe

**Fig. 6:**
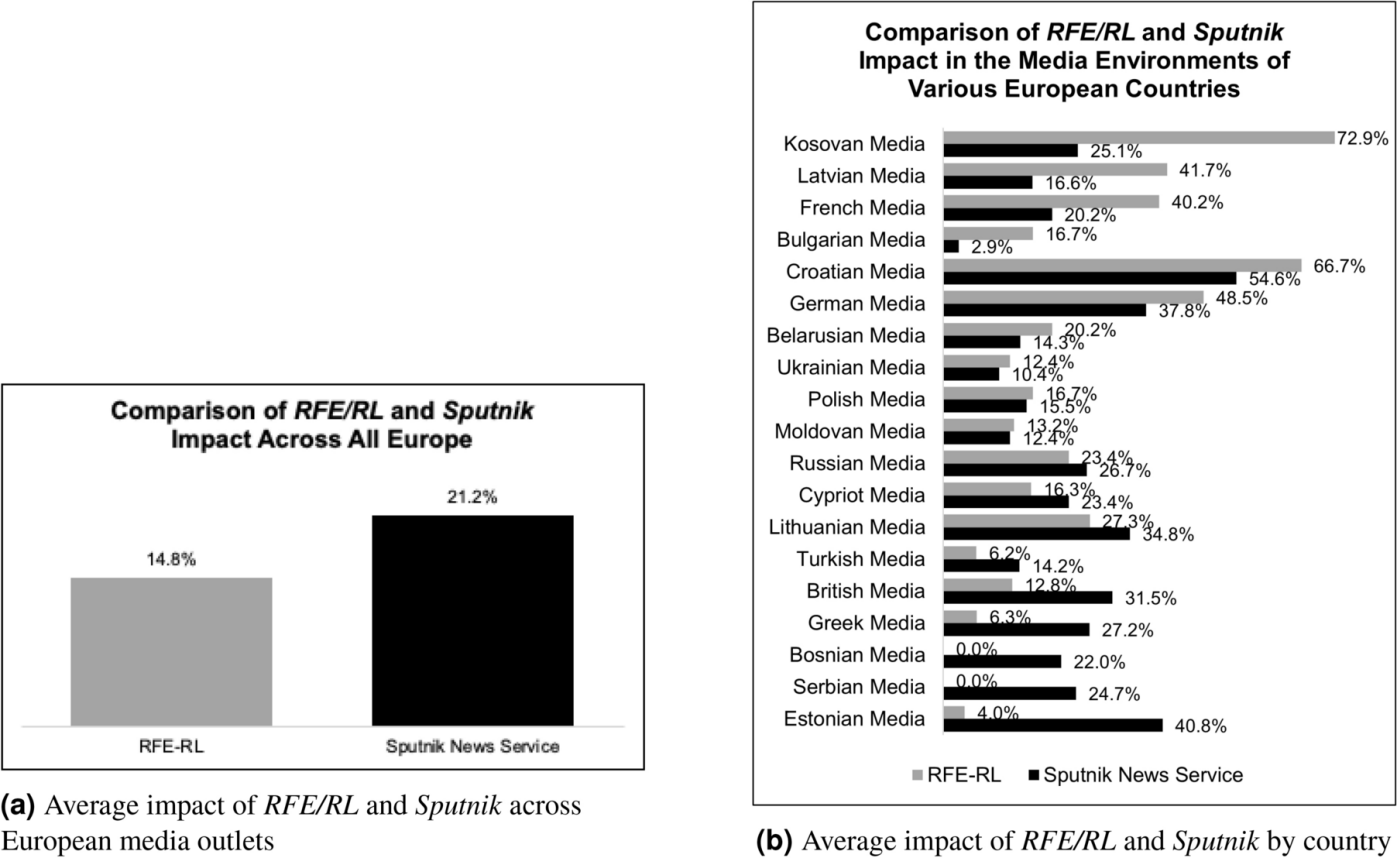
Comparison of *RFE/RL* and *Sputnik News Service* impact in the media environments of various European countries.

Comparing the reach of two state-sponsored media outlets across all countries with outlets represented in our dataset, the Russia-sponsored *Sputnik* has greater overall impact on European media than does the U.S.-sponsored *RFE/RL*, as shown in Figure 6. *RFE/RL* nevertheless has an advantage in the media of the following countries: Bulgaria, Kosovo, Germany, Latvia, Romania, France, and Cyprus. Russian information and influence campaigns are more commonly referenced in the Baltics, the Balkans, and in Eastern Europe. These findings provide a quantifiable measure of one aspect of Russian influence in those regions.

### Case study: the end of the intermediate-range nuclear forces treaty in 2019

One advantage of our approach is we can quantify impacts over any specific topics or range of sentiments. We have already demonstrated the sentiment-based result generation of pro-Russia versus pro-U.S. in the above results based on all topics. Below, we analyze a specific topic: the end of the Intermediate-Range Nuclear Forces (INF) Treaty. In late 2018 and early 2019, we observed Russian state-aligned outlets voicing a consistent messaging narrative on the topic of nuclear treaties: the United States is breaking existing treaties without cause. Russian officials made such statements, and Russian state-affiliated outlets propagated these statements by quoting the officials prolifically to create a narrative of the United States’ destabilizing behavior^23–28^. Conversely, the U.S. position was that Russia designed and deployed weapons that broke the treaty in the previous several years and that the INF was out of date and irrelevant for current security challenges^29,30^. Our network-based approach to estimating influence allows us to understand how certain outlets influence other outlets, thereby propagating narratives voiced in both positions. To avoid unnecessary confounding correlation between the network and the quote outcomes, we construct the influence network using all quotes except those on the nuclear treaty topic. Note, the estimated impact in this case study has higher uncertainty because we have fewer quotes to analyze after restricting our dataset to a single topic.

**Fig. 7:**
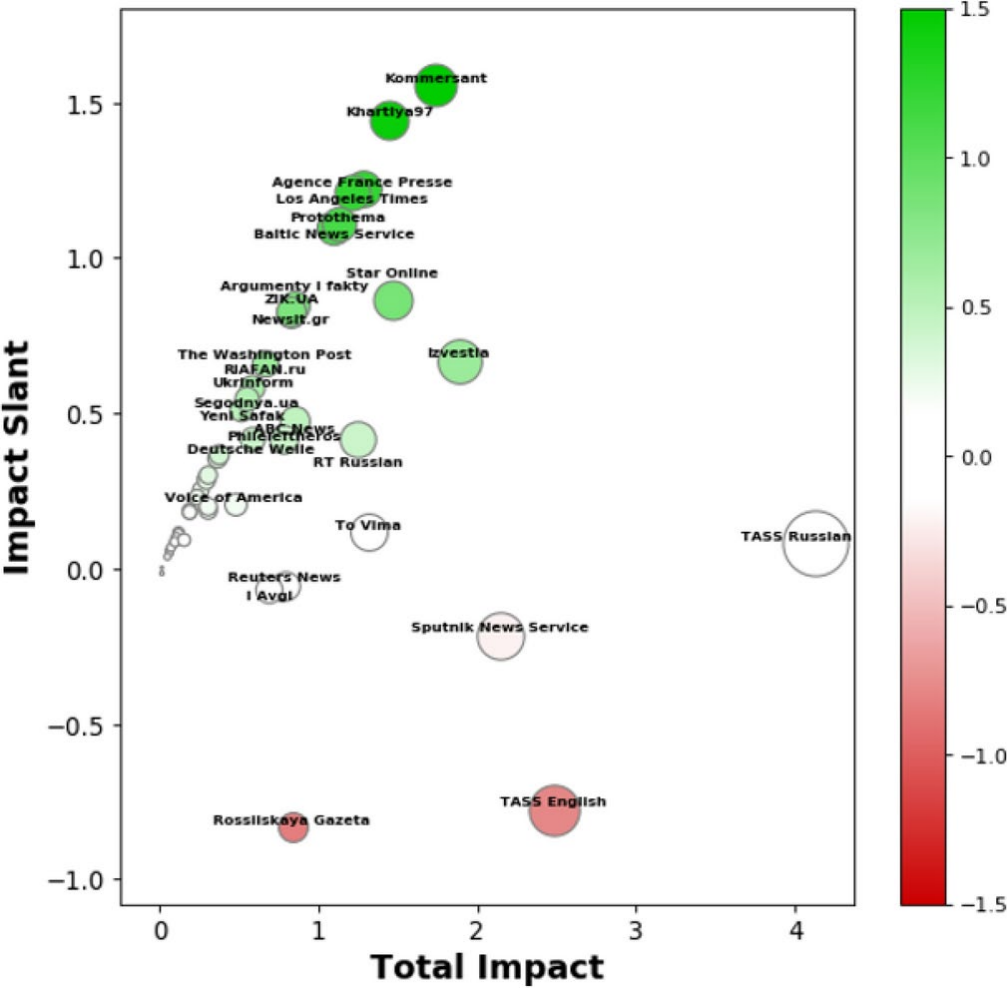
Media outlet impact slant and total impact on the nuclear treaty topic. Outlets are sized by total impact and colored by impact slant. Neutral outlets are white. The more pro-U.S. or pro-Russia slanted an outlet is, the more green or red, respectively. The grey dotted lines indicate positions where all impact came in either the pro-U.S. or pro-Russia slant.

From the overall results on the nuclear treaty topic (Figure 7), one can see that *Rossiiskaya Gazeta* and *TASS English* had the most pro-Russia impact on other outlets. *TASS Russian* had the highest total impact on the topic, meaning it was the most significant determiner of coverage. Strikingly, *TASS Russian’s* impact was rather neutral, which, upon further examination, is a result of other outlets sourcing from *TASS Russian* for U.S. official statements on the nuclear treaty with Russia. One strength of our approach is it allows us to investigate the individual quotes that were influential. If we inspect *Kommersant’s* top followed quotes, for example, we see that they are mostly pro-U.S. *Kommersant* had three times as much pro-U.S. outcome as pro-Russia outcome despite publishing roughly the same number of pro-U.S. quotes as pro-Russia quotes. Upon closer examination, *Kommersant*’s pro-Russia quotes were not picked up by other Russian outlets, which limited *Kommersant*’s pro-Russia impact. This is consistent with *Kommersant*’s network position on the periphery of the Russian state media community in Figure 1.

## Discussion

In developing a quantitative approach to measure agenda setting between outlets by both topic and sentiment, we have identified a method to measure the impact that state-controlled outlets have on other outlets. Using quotations as the unit of analysis, we have shown how Russian state-controlled outlets can create an echo chamber, determining how a topic is covered across the media ecosystem. Our approach also helps us better understand patterns in media outlets’ selection of quotations when covering specific topics. Our results reveal an implicit, obscured level of influence by Russian state media where it sets the parameters for how other outlets report on a topic, regardless of the ownership or declared orientation (e.g. independent, liberal, opposition, etc.) of those outlets. Our results also identify outlets that serve as bellwethers for particular topics.

The implications of these findings on strategic communications and influence operations are significant. From a monitoring and evaluation standpoint, this approach can show the effectiveness of communications campaigns. It can also help independent observers or European governments track the relative performance and influence of outlets like *Sputnik* or *RT* on a European country’s media environment. Editorial boards striving for independence can measure the extent to which their coverage - by topic - is determined by the agenda set from state-controlled media. Such a metric is also of general use to consumers of information who want to know the extent to which a selected source of news or its coverage of a particular topic is influenced by state-controlled media.

For strategic communicators and those studying influence operations alike, this approach offers metrics of performance and effectiveness. Understanding which outlets have impact on a topic-by-topic basis can help in identifying key events for communication such as interviews; for those attempting to understand particular narratives or track disinformation on a topic, this method offers a quantitative approach to estimating causal impact on the broader information ecosystem.

## Methods

### Data collection and labeling

For our corpus, we use data collected from a wide selection of prominent European and Russian traditional media outlets. We collected news articles using a variety of methods such as web scrapers (e.g. Connotate), research and information tools (e.g. Factiva, LexisNexis), and hand clipping of articles. The genre of the corpus is geopolitical news coverage. In collecting the dataset, we filtered for diplomatic, informational, military, and economic content. Although the dataset draws more heavily from countries in Eastern Europe than Western Europe, it includes the top outlets from 24 countries.

To understand the underlying content and sentiments in each news article, we extract all quotations and label each quotation with who the quoted speaker is, a label of the topic based on a discrete set of geopolitical issues, and a label of the sentiment of that quote toward a particular entity. For example, a quote might be labeled with speaker Vladimir Putin, topic “discussing Economic Sanctions,” and sentiment-to-entity “negative to U.S.” The coding was conducted by a team of nine analysts. Sentiment was assigned based on the analysts’ informed insight on the topic at hand and familiarity with the public and political discourse at the time to interpret whether the quote shared criticism of the entity in question or revealed information that is unfavorable to the goals, status, or conditions of the entity in question. The same quote used across different articles had the same sentiment assigned unless outlets truncated or elongated the quote they published in a way that could alter the sentiment.

We also append information about the date of the article, the country of media origin, any geographic references in the quotation, and the outlet where the quote and article appeared. The result is an enriched dataset of 618,328 quotes from 123,396 articles published between May 2018 and October 2019. It includes articles from 454 outlets labeled with one of 167 topics and 418 sentiments. More information on data collection and coding practices can be found in the Supplemental Materials.

### Quotation processing

We start our automated analysis by matching identical quotes. We implement a new method for identifying matching sentences by pairing a transformer language model with density-based clustering. We begin by translating our quote corpus into an embedded space using a pre-trained SBERT model, a language model optimized for semantic textual similarity tasks^31^. To lower the computational load, we reduce the dimensionality of the embeddings and use only the first 70 principal components. Finally, we cluster the transformed embeddings using HDBSCAN such that quotes in the same cluster represent the same quote^32,33^. The classification performance at a representative operating point is a recall of 94% of all matching quotes in the dataset, with precision of 78% correctly matched matches. The full precision-recall curve is provided in the Supplemental Materials. Of the 618,000 original quotes, 367,000 matched another quote in the dataset. Of these matched quotes, 200,000 were published by more than one outlet. After applying our temporal constraint, we have 16,870 instances of one outlet following another outlet’s quote. The median quote is used by three outlets and followed one time by each outlet.

The final step of our quotation processing is to transform these matched quotations into signals of agenda setting. The näive approach would be to count all instances of quote matches where the labeled topic, sentiment, and speaker also match. We improve upon this approach by applying three heuristics that approximate when quote matches may be a signal of influence. First, *potential influence should only be attributed for quotes used after exposure*. Second, *several outlets using the same quote suggests that the quote may be general knowledge*. Finally, *the number of times an outlet follows a quote has diminishing signal*. Discounting for multiple outlets using a quote – as well as repeated uses of a quote – is essential to avoid over-attributing potential influence to outlets employing routine statements. For instance, when Turkey acquired Russian S-400 air defense systems, the U.S. issued a statement that this acquisition jeopardized Turkey’s role in NATO due to the Russian system’s incompatibility with the U.S.-made F-35 fighter jet. In our sample, 57 outlets used this quote, with many repeating it across articles. Without discounting, our model would assign inflated potential influence to outlets that first used this quote, such as Turkey’s *Anadolu News Agency*. This quote is an example of general knowledge and a statement that should be down-weighted in its saliency as a signal of influence between outlets. We model both general knowledge and diminishing signal as the monotonically increasing and concave square root function. A more thorough explanation of this process can be found in the Supplemental Materials. The following equation incorporates the above heuristics to transform a set of quotes *q* into saliency-weighted potential quote influence of outlet *i* on outlet *j*:

1 $$\begin{aligned} k_{ij} = \sum _q \frac{1}{\sqrt{\# \text { of outlets using }q}} \times \sqrt{\# \text { of times }j\text { uses }q} \times \frac{\# \text { of times }j\text { uses }q\text { after }i\text {'s first use}}{\# \text { of times }j\text { uses }q} \end{aligned}$$

### Network construction and causal impact estimation

Next, we construct an influence network between media outlets using these saliency-weighted potential quote influences. Each node in the network represents a media outlet, with directed and weighted edges representing potential influences between them, represented by the adjacency matrix *A*. Because actual influence is not directly observable, each network edge is modeled as a random variable with Poisson prior distribution parameterized by the observed saliency-weighted quote influence, $a_{i,j} \sim \text {Poisson}(k_{i,j})$, for realistic network interactions^34^.

The final stage of the methodology performs causal inference to estimate the causal impact between outlets using the saliency-weighted quote influence and the constructed influence network prior. This causal inference framework over networks was developed by Kao and Rubin and demonstrated on social media networks by Smith et al^16,20,35,36^. This paper is its first demonstration on news media networks. The causal impact of outlet *i* on outlet *j* is explicitly captured in the causal estimand:

2 $$\begin{aligned} {\zeta _{i,j} \buildrel \textrm{def}\over = Y_j (\varvec{z}_{i+}, \varvec{A}) - Y_j (\varvec{z}_{i-}, \varvec{A})} \end{aligned}$$

where $Y_j (\varvec{z}_{i+}, \varvec{A})$ represents potential outcome on outlet *j*’s saliency-weighted quotes, with the binary vector $\varvec{z}_{i+}$ denoting outlet *i* being the quote source on the influence network represented by the directed and weighted adjacency matrix $\varvec{A}$. Outlet *i*’s causal impact on outlet *j* is established by subtracting the counterfactual outcome of outlet *j*’s saliency-weighted quotes in the absence of outlet *i* as the source, $Y_j (\varvec{z}_{i-}, \varvec{A})$. In summary, the difference between these two potential outcomes represents outlet *i*’s causal impact on outlet *j*’s outcome. The first potential outcome $Y_j (\varvec{z}_{i+}, \varvec{A})$ is observed as outlet *i*’s saliency-weighted quote influence on outlet *j*, in Equation 1. However, the counterfactual outcome is not directly observable. They are imputed using a Poisson generalized linear mixed model (GLMM) fitted to the observed outcome $Y_j (\varvec{z}_{i+}, \varvec{A})$ on each outlet *j*.

The key challenge to estimating the causal impact between outlets on a network is the presence of social confounders such as homophily, where outlets nearby each other on the influence network share similar reporting behaviors. In our influence network, outlets may follow quotes for reasons *other than* outlet influence; for example, outlets may share a geopolitical focus or origin country that shapes similar reporting. Thus, our causal inference approach must disentangle the causal effects of network influence from such confounder effects. We account for these confounder effects via covariate adjustment by including them in the Poisson GLMM potential outcome model. This outcome model captures the effects of each *n*-hop exposure to the quote source via the influence network, network confounders such as node degrees and outlet community membership, and heterogeneity between outlets. Outlet community membership is estimating by fitting the influence network to a degree-corrected stochastic blockmodel^37^. These community memberships capture the geopolitical proximity between outlets. The Poisson distribution models the saliency-weighted quote outcomes as Poisson processes. The Poisson GLMM uses the canonical log-link function and includes linear predictor coefficients ($\tau , \gamma , \beta , \mu$), corresponding to the source indicator $z_j$, n-hop exposures $s_j^{(n)}$, the covariate vector $\varvec{x_j}$, and the baseline outcome:

3 $$\begin{aligned} \begin{aligned}&{Y_j(\varvec{z}, \varvec{A}) \sim \text {Poisson}(\lambda _j)}\\&{\log (\lambda _j) = \tau z_j + \sum _{n=1}^{N_\text {hop}} s_j^{(n)} \tau \prod _{k=1}^{n} \gamma _k + \varvec{\beta }^T \varvec{x}_j + \mu + \epsilon _j} \end{aligned} \end{aligned}$$

In the five effect terms, $\tau z_j$ represents the primary effect of the quote source, $\sum _{n=1}^{N_\text {hop}} s_j^{(n)} \tau \prod _{k=1}^{n} \gamma _k$ represents the accumulative network influence effect from each *n*-hop exposure $s_j^{(n)}$ to the source, $\gamma _k$ (between 0 and 1) represents how quickly the effect decays over each additional *k*th hop, $\varvec{\beta }^T \varvec{x}_j$ is the effect of the unit covariates $x_j$ of network confounders. Lastly, $\mu$ is the baseline effect on each outlet, and $\epsilon _j \sim \text {Normal}(0, \sigma ^2_\epsilon =.1)$ provides independent and identically distributed variation for heterogeneity between the outlets. More details on the causal impact estimation can be found in the Supplemental Materials.

## Supplementary Information

Supplementary Information.

## Data Availability

A preprocessed version of the dataset sufficient for replication of our results can be found at the Mendeley Repository.
