# Supplementary material for: Exposing the obscured influence of state-controlled media via causal inference of quotation propagation
Source: Sci Rep. 2025 Jan 7;15:1110. doi: 10.1038/s41598-024-78586-x (PMC11707100; doi:10.1038/s41598-024-78586-x)
Supplement: Supplementary file 1 — Supplementary Information. [file 41598_2024_78586_MOESM1_ESM.pdf]

The following supplemental sections primarily serve to provide a more detailed explanation of our methodology such that future researchers can replicate this work. We follow our methodology supplement with additional results.

Methods

Our novel quantitative approach measures the causal impact between media outlets as they propagate quotations with specific sentiments on the topic of interest. Beyond the initial data curation process, this approach is automated with state-of-the-art text analytics and novel causal inference on networks. Our methodology has four main components, as shown in Figure 1. First, we collect a dataset of quotes pulled from articles from prominent European digital media outlets and hand-label them with context-based sentiment and topic. Then, we extract matching quotes from the dataset using a novel and automated method by mapping each quote to a semantic space using the transformer model SBERT and then identifying matching quotes through clustering with HDBSCAN. Matching quotes are weighted by salience, such that quotes with better signal of influence are weighted more than those with lower signals. Next, we construct an influence network using each media outlet as a node and connecting edges based on historical *quote following* (when an outlet publishes a quote identical to one from another outlet). Finally, we apply a novel network causal inference method to quantify causal impact between outlets.

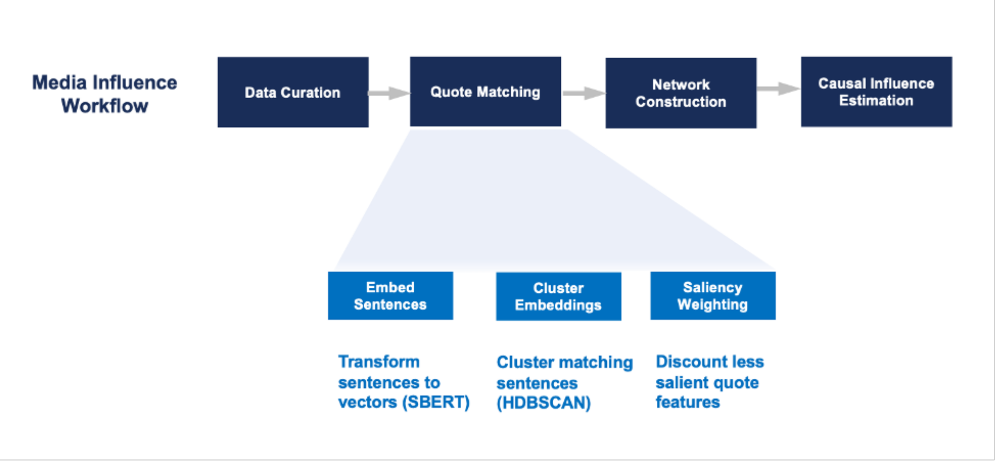

Figure 1. Data-driven methodology for semi-automated media influence estimation

Suitability of Quotes as Instrument

Our paper is unique among the literature for its use of quotations as the instrument for influence. This section expands on our justifications for using quotations. When an outlet features a quotation within an article, a decision is made by the authors and editors to feature this particular voice as a means to elucidate details of the article's topic, or relevant opinions that provide context and insight. These quotations form the syntactical unit of the subsequent news coverage that readers consume [1]. The authors have extracted quotations—to include paraphrased quotes—as a means to validly separate discrete units of content. Quotations are particularly well suited as valid syntactical units of traditional media with limited distortion when separated from the article's context [2]. Examining the decisions of whom to quote highlights how outlets influence what issues news consumers pay attention to and what positions they consider, a process referred to as *agenda setting* [3]. In topics with competing narratives, the choice of whom to quote, which quotations to highlight, and how many voices from each side of an argument to include will determine the media coverage to which the reader is exposed, thus justifying quotations as the basic unit of analysis in outlet coverage leaning and influence.

Quotes are a suitable unit of analysis from a practical standpoint, as well. The diffusion of quotes can be reliably and automatically detected. While an outlet may excerpt or otherwise change a quote, the quote remains recognizable.

Data Collection and Labeling

For our corpus we use data collected from a wide selection of the most prominent paid and unpaid European and Russian traditional media outlets based on third-party services like Alexa.com and SimilarWeb that track the popularity of webpages. The genre of the corpus is geopolitical news coverage. Content was aggregated using standardized search strings filtering for diplomatic, informational, military, and economic content.[1] The data set draws more heavily from countries in Eastern

[1]One search string example is: ("NATO" OR (("USA" OR "America" OR "European Union" OR "Latvia" OR "Lithuania" OR "Estonia" OR "Baltics" OR "Ukraine" OR "Poland" OR "Balkans" OR "Turkey" OR "Georgia" OR "Greece" OR "Serbia" OR "Croatia" OR "Montenegro" OR "Bosnia" OR "Finland" OR

Europe than Western Europe but includes the most prominent outlets in 24 countries. Though news consumption preferences vary considerably within every country in Europe, the dataset has selected the most prominent outlets in select countries to approximate the news coverage of geopolitical issues that an average European is exposed to on a regular basis. As such, we have included a small number of U.S.-based outlets to capture the borderless nature of media consumption in Europe. For those outlets based in Russia, the data set includes media affiliated with the Russian government as well as media that is not.

To understand the underlying content and sentiments in each news article, we extract all quotations and label each quotation with who the quoted speaker is, a label of the topic based on a discrete set of geopolitical issues, and a label of the sentiment of that quote toward a particular entity. For example, a quote might be labeled with speaker Vladimir Putin, topic "discussing Economic Sanctions," and sentiment-to-entity "negative to U.S." The coding was conducted by a team of nine analysts, three of whom coded Russian-language content in natural language and six of whom coded machine-translated English content of all other ingested European languages. The analysts assigned metadata tags such as directional sentiment (e.g. Positive to the U.S., Neutral to Russia, Negative to NATO, etc.) and topic (e.g. NATO Exercises, Missile Defense/Deployment, Russian Military Deployments, etc.) to each extracted quote from the ingested articles using a codebook that provided guidelines for what topics to use in a given circumstance. For instance, the guidance for assigning the topic "Nuclear Cooperation" reads as follows: "Use for statements regarding U.S.-Russia nonproliferation cooperation and general cooperation on nuclear security issues." Only one topic and one sentiment could be assigned to each quote. Sentiment was assigned based on the analysts' informed insight on the topic at hand and familiarity with the public and political discourse at the time to interpret whether the quote shared criticism of the entity in question or revealed information that is unfavorable to the goals, status, or conditions of the entity in question. The same quote used across different articles had the same sentiment assigned unless outlets truncated or elongated the quote they published in a way that could alter the sentiment.

Given the numerous topics (144), directional sentiment entities (165), and coders (9), reliably achieving agreement among the coders presented a challenge. We took a designated coder approach to inter-coder reliability in which all analysts' coding was measured against one individual standard for agreement. To aid in this process, a comprehensive selection and hiring process was conducted to ensure that analysts had an appropriate level of understanding of the geopolitical focus of the dataset. All analysts were college-educated primarily in the fields of Political Science, International Relations, and International Conflict. Some had specific expertise regarding the Russia-Ukraine conflict, the NATO relations, or the Balkan region. Several of the Russian-enabled analysts previously lived in Russia, bringing them closer to the topic of analysis and expediting the training process of understanding project methodology (topics and sentiments), identifying political media affiliation, rhetoric and narratives used by various actors, and deciphering the true meaning and sentiment of each quotation. All content enriched by new analysts during their first month was assessed by a quality assurance team (which represents the designated coder) during multi-hour meetings with the analyst to review, discuss, and if necessary correct metadata assignments according to the methodology. This probationary period lasted up to 5 weeks, and new analysts were instructed not to add coded content to the finalized dataset until it was reviewed by the quality assurance team. This process ensured a complete learning process for the analyst to achieve consistency and agreement among the coders with the designated coder according to the established methodology in the codebook. Following the probationary period, each week a 10–15% sample of every coder's output quota was analyzed for agreement with the designated coder. Metrics of agreement included sources, sentiment, locations, and other metadata fields. The quality assurance team met with each analyst to inform them of the results of the review process, to discuss ways to improve efficiency and agreement, and to identify metadata assignments that stood in need of correction.

Quotation Matching

We used HDBSCAN to cluster quotes such that a cluster indicated a matched quote. We optimized and tested HDBSCAN parameters using a hand-labeled truth set of 1,668 quotes. Results from our clustering optimization can be seen in Figure 2, with specific examples provided in Table 1. At the optimal HDBSCAN parameters, we achieved 94% recall and 78% precision on our task of clustering quotes. In general, false positives tend to occur with quotes that use jargon, such as the quote discussing S-400s and F-35s. They also occur with subjects where there are not many quotes; these quotes may end up evenly-spaced in the embedded space and the density-based clustering will group them together. False negatives tend to occur on difficult paraphrase matching, or in areas where the same quote is used in slightly different form many times and the clustering algorithm fails to merge two nearby clusters.

While there are no directly comparable benchmarks in the literature, we can compare our results to "paraphrase identification" results. Wang et al. report 89% basic accuracy on paraphrase identification, where accuracy is the sum of true positives and true negatives divided by all labels [4]. We demonstrate 99.4% accuracy on our task. Admittedly, our quote matching task is less challenging than paraphrasing; while some of quote matching is paraphrase identification, many of the quotes are near-exact

"Azerbaijan" OR "Armenia") AND ("rocket" OR "missile" OR "missiles" OR "corruption" OR "nuclear" OR "gas" OR "gazprom" OR "humanitarian aid" OR "human rights activist" OR "human rights" OR "refugees" OR "protest" OR "demonstration" OR "freedom of the press" OR "terrorism" OR "ISIS" OR "Arctic" OR "intercept" OR "interception" OR "exercises" OR "elections" OR "aircraft carrier" OR "military")))

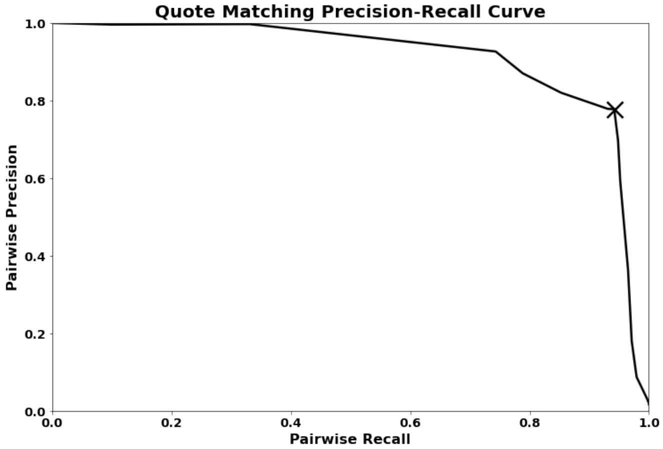

Figure 2. Precision recall curve for quote matching clustering. At the X (our optimum), we achieve 78% precision and 94% recall. That is, 78% of identified quote matches are true matches and we capture approximately 94% of all quote matches.

matches. Mellace et al. proposed a similar sentence matching pipeline using SBERT and DBSCAN; they analyze novels and cluster sentences that captured similar relations between characters, such as characters speaking to or smiling at other characters [5].

Saliency-weighted Quote Influence Heuristics

We apply three heuristics to transform sets of matched quotes into saliency-weighted quote influence. This section explains our assumptions more precisely. First, *potential influence should only be attributed for quotes used after exposure*. We apply this heuristic by scaling our value by the proportion of matched quotes that came after possible exposure. Second, *several outlets using the same quote suggests that the quote may be general knowledge*. We assume that the more outlets there are using the quote, the likelier it is to be general knowledge and the less signal the instance of quote following contains. Thus, we discount by some function of the number of outlets that used the quote. We represent this heuristic by function $g_1(x)$, a monotonically increasing and concave function. The final heuristic is *diminishing signal on the number of times an outlet uses a quote*. More plainly, the first time an outlet uses a quote is more informative than the tenth time. We model this heuristic as a monotonically increasing and concave function, $g_2(x)$. Let Q be the set of quotes outlet i used, S_q be the number of outlets that used quote q and let $T_{q,i}$ be the time outlet j used quote q. The following equation incorporates the above heuristics to transform the sets of matched quotes into saliency-weighted quote influence of outlet i on outlet j:

$$\kappa_{ij} = \sum_{q \in Q_i \cap Q_j} \frac{1}{g_1(S_q)} g_2(|q \in Q_j|) \frac{\left|\{q \in Q_j \ : \ t_{q,i} \geq \min(t_q)\}\right|}{\sqrt{|q \in Q_j|}} \tag{1}$$

Inside the summation, the first term models the general knowledge discount, the second term models the diminishing signal on the number of times an outlet uses a quote, and the third term is the fraction of quotes after initial exposure. For this analysis, we substitute the square root function as our monotonically increasing and concave function ($g_1(x) = g_2(x) = \sqrt{x}$), which gives us the following equation:

$$\kappa_{ij} = \sum_{q \in Q_i \cap Q_j} \frac{1}{\sqrt{S_q}} \sqrt{|q \in Q_j|} \frac{\left|\{q \in Q_j \ : \ t_{q,i} \geq \min(t_q)\}\right|}{\sqrt{|q \in Q_j|}} \tag{2}$$

Figure 3 provides an example of how saliency-weighting is applied on a potential quote influence of *AFP* on *Sputnik*.

Potential Outcome Model for Causal Impact Estimation

The next step is to pair the processed quotes with a network causal inference framework [6, 7]. The saliency-weighted quote influence only represents *potential influence* between the outlets. Actual causal impact of outlet i on outlet j is explicitly captured in the causal estimand:

$$\zeta_{i,j} \stackrel{\text{def}}{=} Y_j(\boldsymbol{z}_{i+}, \boldsymbol{A}) - Y_j(\boldsymbol{z}_{i-}, \boldsymbol{A}) \tag{3}$$

	Source	Quote 1	Quote 2
**True Positives**	Russian Embassy Statement	Moscow "repeatedly answered the propaganda" calls of the "West" to release those under investigation, defendants or convicts in accordance with Russian law for serious crimes. *RT Russian*, 2018-06-19	Russia has repeatedly and unequivocally responded to propaganda “calls” from the West for the release of certain suspects, defendants or convicts in accordance with our legislation for various serious crimes. *TASS Russian*, 2018-06-17
	Maxim Oreshkin	There is road-building machinery and a number of other items that Russia imports. *Reuters News*, 2018-06-19	Among the goods in respect of which these duties may be introduced, "road construction equipment and a number of other elements that Russia imports." *Interfax*, 2018-6-20
	U.S. Government Statement	Russia’s 9M729 missile violates the INF Treaty and Moscow’s assurances that the missile’s range is within the limits allowed by the agreement. *Sputnik News Service*, 2019-06-28	Moscow in violation of the contract due to the presence of the 9M729 missile, the flight range of which allegedly exceeds acceptable standards. *Izvestia*, 2019-07-26
**False Positives**	Hulusi Akar	Works are under way to protect and protect the rights arising from international law and agreements, and that they will do their best to protect the rights of the blue citizens. *Karar Online*, 2018-12-20	When we talk about Blue Homeland, we mean a 462km area that includes both the sea and the sky. We have rights set out in international treaties and expect our interlocutors and neighbors to respect them. In this context, we expect to move forward. *To Vima*, 2019-08-12
	Unnamed U.S. Officials	Suggested Turkey buy the U.S. Patriot missile system rather than the S-400, arguing it is incompatible with NATO systems and is a threat to the F-35 fifth-generation stealth aircraft. *Daily Sabah*, 2019-05-03	The government of US President Donald Trump still intends to impose sanctions on Turkey and exclude it from the F-35 construction program if it finally purchases the S-400. *Protothema*, 2019-07-04
**False Negatives**	Mike Pompeo	The United States calls on Russia to respect the principles to which it has long claimed to adhere and to end its occupation of Crimea. *CNN*, 2018-07-25	The U.S. would hold to its long-standing principle of refusing to recognize Kremlin claims of sovereignty over territory seized by force in violation of international law. And he called for Russia to respect principles it claims to respect and "end its occupation of Crimea. *Los Angeles Times*, 2018-07-25
	Mevlut Cavusoglu	We must first clarify what we will negotiate at the informal five-day conference and then supplement the content of the terms of reference. *To Thema*, 2019-09-15	Stated that, for this reason, there is a need for a five-day informal conference, so that the sides can clarify what will be discussed next in the Cyprus issue. Then the terms of reference will be clear. *I Kathimerini*, 2019-09-09

Table 1. Examples of quote matches, successful and unsuccessful. True positives were quotes that matched correctly. False positives are quotes that matched, but should not have because they are actually different quotes. False negatives are quotes that did not match, but should have because they are the same quote.

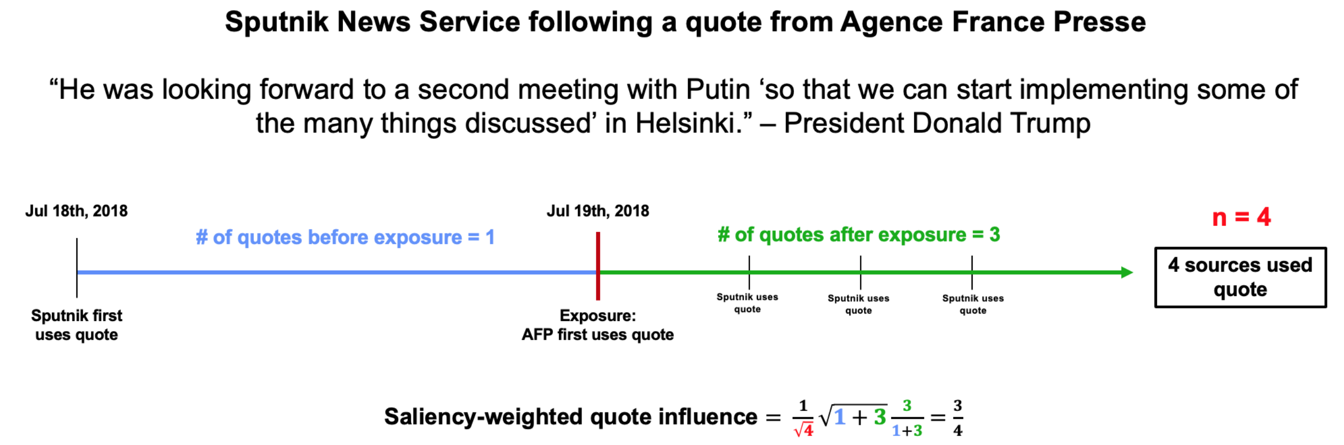

Figure 3. Saliency-weighted quote influence example. *Sputnik* first used this quote on July 18th. *AFP* first used it the next day on July 19th. *Sputnik* then used the quote three more times. So, three out of four instances of *Sputnik* using the quote came after exposure to *AFP*. A total of four outlets used the quote. For this specific quote, *AFP* has a saliency-weighted potential quote influence of $\frac{3}{4}$ on *Sputnik*.

where $Y_j(\boldsymbol{z}_{i+},\boldsymbol{A})$ represents potential outcome on outlet j's saliency-weighted quotes, with the binary vector $\boldsymbol{z}_{i+}$ denoting outlet i being the quote source on the influence network represented by the directed and weighted adjacency matrix $\boldsymbol{A}$. Outlet i's causal impact on outlet j is established by subtracting the counterfactual outcome of outlet j's saliency-weighted quotes in the absence of outlet i as the source, $Y_j(\boldsymbol{z}_{i-},\boldsymbol{A})$. In summary, the difference between these two potential outcomes represents outlet i's causal impact on outlet j's outcome. The first potential outcome $Y_j(\boldsymbol{z}_{i+},\boldsymbol{A})$ is observed as outlet i's saliency-weighted quote influence on outlet j, in Equation 2. However, the counterfactual outcome is not directly observable. They are imputed using a Poisson generalized linear mixed model (GLMM) fitted to the observed outcome $Y_j(\boldsymbol{z}_{i+},\boldsymbol{A})$ on each outlet j.

This outcome model captures the effects of each n-hop exposure to the quote source via the influence network, network confounders such as node degrees and node community membership, and heterogeneity between the outlets. The Poisson distribution models the saliency-weighted quote outcomes as Poisson processes. Practically, the saliency-weighting makes the observed outcomes non-discrete so they need to be rounded before being fitted to the model. To reduce the rounding effect and increase the resolution of the Poisson-distributed outcome, the observed outcomes are multiplied by a scalar of ten before rounding. This makes a unit of causal impact in this paper equal to 0.1 saliency-weighted quote defined in Equation 2. The Poisson GLMM uses the canonical log-link function and includes linear predictor coefficients (τ,γ,β,μ), corresponding to the source indicator z_j, n-hop exposures $s_j^{(n)}$, the covariate vector $\boldsymbol{x}_j$, and the baseline outcome:

$$\begin{aligned} Y_j(\boldsymbol{z},\boldsymbol{A}) &\sim \text{Poisson}(\lambda_j) \\ \log(\lambda_j) &= \tau z_j + \sum_{n=1}^{N_\text{hop}} s_j^{(n)} \tau \prod_{k=1}^{n} \gamma_k + \boldsymbol{\beta}^T \boldsymbol{x}_j + \mu + \varepsilon_j \end{aligned} \tag{4}$$

In the five effect terms, τz_j represents the primary effect of the quote source, $\sum_{n=1}^{N_\text{hop}} s_j^{(n)} \tau \prod_{k=1}^{n} \gamma_k$ represents the accumulative network influence effect from each n-hop exposures $s_j^{(n)}$ to the source, γ_k (between 0 and 1) represents how quickly the effect decays over each additional kth hop, $\boldsymbol{\beta}^T\boldsymbol{x}_j$ is the effect of the unit covariates x_j of network confounders including node degrees and node community membership (fitting the influence network to a blockmodel with five communities). These confounders are accounted for through covariate adjustment to disentangle actual causal impact from effects of homophily (birds of a feather flock together) and vertex degrees. Lastly, μ is the baseline effect on each outlet, and $\varepsilon_j \sim \text{Normal}(0,\sigma_\varepsilon^2 = .1)$ provides independent and identically distributed variation for heterogeneity between the outlets. The amounts of social exposure at the nth hop are determined by $\boldsymbol{A}^{T^n}\boldsymbol{z}$. This captures quote diffusion via all exposure paths to the quote source over the influence network. Diminishing return of additional exposures is modeled using (elementwise) log-exposure, $s^{(n)} = \log(\boldsymbol{A}^{T^n}\boldsymbol{z}+1)$.To account for the uncertainty of the influence network $\boldsymbol{A}$, it is jointly estimated with the model parameters (τ,γ,β,μ) through Markov Chain Monte Carlo (MCMC) and Bayesian regression.

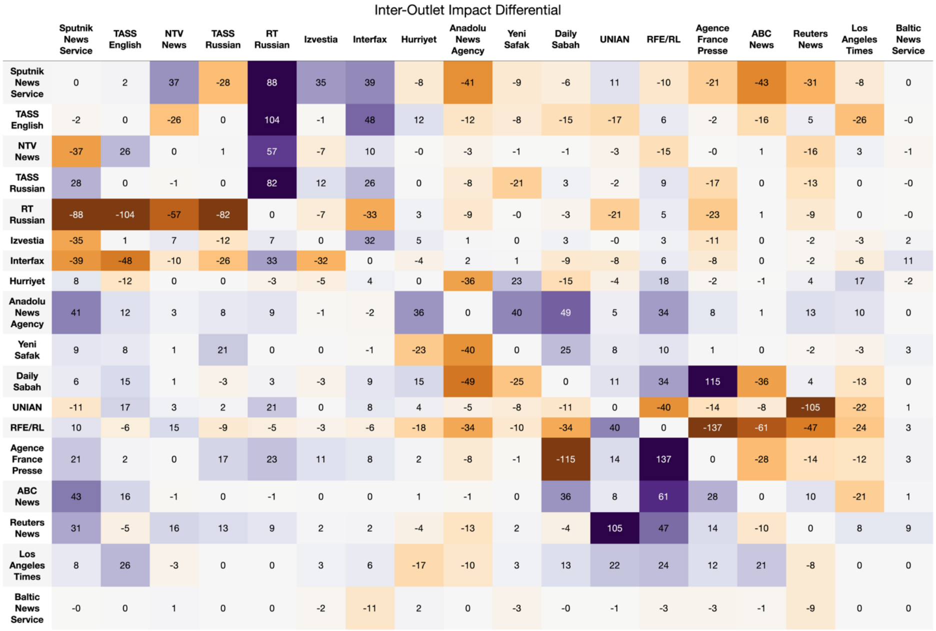

Figure 4. Total impact differentials of the top 19 outlets. These values represent the net impact of the row outlet on the column outlet. Negative, brown values mean the column outlet had more impact on the row outlet, while positive, purple values mean the row outlet had more impact than the column outlet. White, small magnitude values suggest symmetrical impact.

Results

Directionality of Influence and Impact Differentials

We can also examine the directionality of influence by looking at total impact differential (Figure 4). An outlet i's total impact differential with respect to outlet j is defined by the impact of i on j minus the impact of j on i. In general, impact is symmetric with outlet i influencing outlet j about as much as outlet j influences outlet i; this leads to an impact differential near zero, as is the case between *Sputnik* and *TASS English*. However, there are some outlets that tend to have asymmetric influence. *RT Russian* stands out as a follower, or an outlet that is influenced more than it influences. Looking at the row for *RT Russian* in Figure 4, many of the values in the Russian area are quite negative and brown, which indicates Russian outlets exert more influence on *RT Russian* than *RT Russian* exerts on them. Equivalently, the column for *RT Russian* is positive and blue. In the other direction, some outlets are influencers and tend to influence more than they are influenced. One example of an influencer outlet might be *Sputnik* or *Anadolu News Agency*, who generally demonstrate net influence on other outlets in their media ecosystem. While *Sputnik* is only an influencer in the Russian sphere, *Anadolu News Agency*'s positive differential persists with non-Turkish outlets as well, although to a lesser extent. *RFE/RL* is more often influenced by Western outlets such as *Agence France Presse*, *ABC News*, and *Reuters News*. This tracks well with *RFE/RL*'s mission of bringing Western-style reporting to other countries, not being a prominent outlet in the Western media environment. That Turkish outlet *Daily Sabah* has high asymmetric influence over *Agence France Presse* could suggest *Agence France Presse* relies on *Daily Sabah* in reporting on matters pertaining to Turkey.

INF Case Study Extended

Our paper references *Kommersant* as an outlet that surprisingly has 3x as much pro-U.S. outcome as pro-Russia outcome despite publishing roughly the same number of pro-U.S. quotes as pro-U.S. quotes. Upon closer examination, *Kommersant*'s pro-Russia quotes were not picked up by other Russian outlets, which limited *Kommersant*'s pro-Russia impact. Figure 5 displays the top few quotes, along with other relevant quote following statistics.

Relationship Between Volume and Impact

One relevant question considers the relationship between quotes and impact: do outlets that publish frequently have the most impact? Moreover, what is the connection between quote slant (how many pro-U.S. quotes you publish versus pro-Russia quotes) and impact slant? Figure 6 examines this relationship. The left plot demonstrates that outlets that published more quotes were generally more impactful. *Sputnik News Service*, the most impact outlet, also published the most quotes. However, the correlation is not perfect. Consider *RT Russian*, which had over twice as many quotes as *Reuters News* and *Agence France*

Source Name	Publication Date	Quote Position	Following Media Name	Following Date	# Quote Uses After Exposure	# Quote Uses Before Exposure	# Sources Using Quote	Outcome	Kommersant Quote	Following Quote
Rus Kremlin Statement	2018-05-09	Negative to U.S.	Vedomosti.ru	2018-05-09	2	0	1	28.28	Deep concern was expressed regarding this decision and the importance of this document was once again emphasized."	Deep concern was expressed in connection with such a decision and once again the importance of this document was emphasized,"
Mike Pompeo	2019-08-02	Negative to Russia	Izvestia	2019-08-14	2	0	1	28.28	the blame lies exclusively with Russia.	that the blame for this lies solely with Russia
Mike Pompeo	2018-12-04	Negative to Russia	Sputnik News Service	2018-12-20	50	0	42	21.82	The United States plans to stop its obligations under the agreement on the elimination of intermediate and shorter-range missiles (INF Treaty) if Russia does not return to its fulfillment in 60 days.	the United States would suspend its adherence to the INF Treaty within 60 days unless Russia returned to full compliance with the agreement.
Donald Trump	2018-10-22	Negative to Russia	TASS English	2018-12-22	80	10	63	21.25	That the US will quit the INF Treaty, as Russia violates the treaty	said on October 20 that Washington would withdraw from the INF Treaty because Russia was violating the terms of the agreement.
U.S. Government Statement	2018-12-04	Negative to Russia	TASS English	2019-08-05	47	1	43	20.69	Moscow violates the agreement.	its actions were provoked by Russia's refusal to comply with the American ultimatum-like demand to eliminate the new 9M729 cruise missiles, which Washington and its NATO allies believe to violate the INF Treaty
Mike Pompeo	2018-12-04	Negative to Russia	TASS English	2019-02-04	44	0	42	20.47	The United States plans to stop its obligations under the agreement on the elimination of intermediate and shorter-range missiles (INF Treaty) if Russia does not return to its fulfillment in 60 days.	Washington suspends the implementation of its obligations under the INF Treaty on February 2, and half a year later will stop being its participant if Russia does not make any steps to ensure a return to the implementation of the obligations under the treaty

Figure 5. Examples of top followed quotes for *Kommersant*. Many of the top quotes were negative to Russia, which explains why *Kommersant* was pro-U.S. over the nuclear treaty topic.

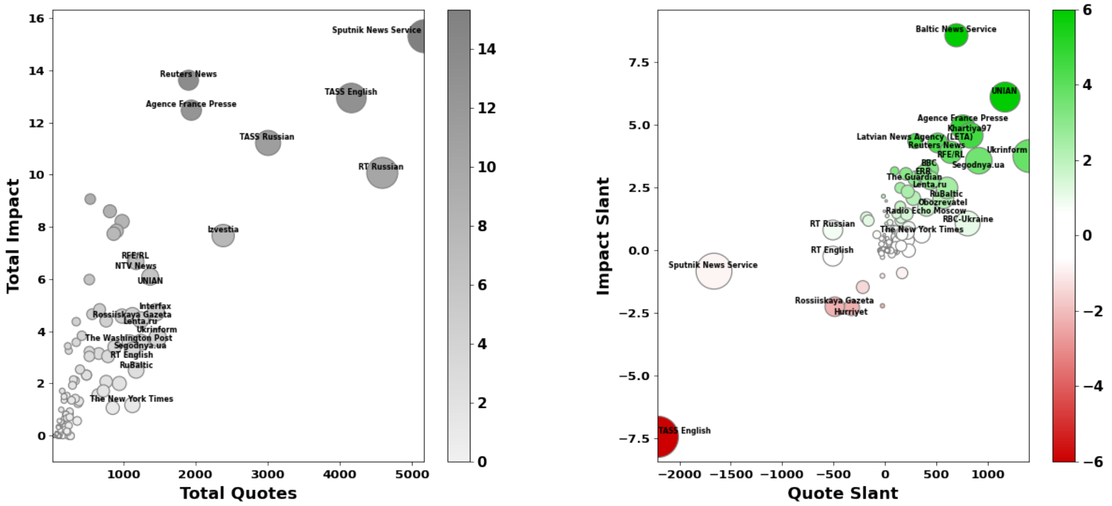

Figure 6. Relationship between quotes and impact. The left figure shows the relationship between total quotes and impact, while the right figure shows the relationship between quote slant and impact. Points are sized according to the x axis and colored according to the y axis. Pro-Russia slant is indicated as negative values and pro-U.S. positive.

Presse, yet had less impact. There is considerable variation in total impact among outlets with the same number of quotes, demonstrating the role that causal inference on networks play to more accurately quantify actual impact from simple activity count statistics.

A similar story holds when we compare impact slant to quote slant. There is a linear relationship between the two, but there is significant variation in impact slant among outlets with similar quote slants. *Sputnik News Service*, for example, is fairly neutral despite very pro-Russia quote slant, which can be explained by its pro-U.S. quotes being generally more impactful and propagated by Western media outlets, shown in Figure 6 of the main paper.

References

1. Krippendorff, K. *Content Analysis: An Introduction to the Methodology* (SAGE, 1980).

2. Fico, F. G., Lacy, S. & Riffe, D. A content analysis guide for media economics scholars. *J. media economics* **21**, 114–130 (2008).

3. McCombs, M. E. & Shaw, D. L. The agenda-setting function of mass media. *Public Opin. Q.* **36**, 176–187 (1972).

4. Wang, Z., Hamza, W. & Florian, R. Bilateral multi-perspective matching for natural language sentences. *arXiv preprint arXiv:1702.03814* (2017).

5. Mellace, S., Vani, K. & Antonucci, A. Relation clustering in narrative knowledge graphs (2020). 2011.13647.

6. Kao, E. K. *Causal inference under network interference: A framework for experiments on social networks*. Ph.D. thesis, Harvard University (2017).

7. Smith, S. T. *et al.* Automatic detection of influential actors in disinformation networks. *Proc. Natl. Acad. Sci.* **118**, e2011216118 (2021).
